# Multifactorial genetic control and magnesium levels govern the production of a *Streptomyces* antibiotic with unusual cell density dependence

**DOI:** 10.1128/msystems.01368-23

**Published:** 2024-03-11

**Authors:** Hindra ­, Marie A. Elliot

**Affiliations:** 1 Institute of Infectious Disease Research and Department of Biology, McMaster University, Hamilton, Ontario, Canada; Universiteit Leiden, Leiden, the Netherlands

**Keywords:** *Streptomyces*, antibiotic, regulation, cell density, phosphate, magnesium

## Abstract

*Streptomyces* bacteria are renowned both for their antibiotic production capabilities and for their cryptic metabolic potential. Their metabolic repertoire is subject to stringent genetic control, with many of the associated biosynthetic gene clusters being repressed by the conserved nucleoid-associated protein Lsr2. In an effort to stimulate new antibiotic production in wild *Streptomyces* isolates, we leveraged the activity of an Lsr2 knockdown construct and successfully enhanced antibiotic production in the wild *Streptomyces* isolate WAC07094. We determined that this new activity stemmed from increased levels of the angucycline-like family member saquayamycin. Saquayamycin has both antibiotic and anti-cancer activities, and intriguingly, beyond Lsr2-mediated repression, we found saquayamycin production was also suppressed at high density on solid or in liquid growth media; its levels were greatest in low-density cultures. This density-dependent control was exerted at the level of the cluster-situated regulatory gene *sqnR* and was mediated in part through the activity of the PhoRP two-component regulatory system, where deleting *phoRP* led to both constitutive antibiotic production and *sqnR* expression. This suggests that PhoP functions to repress the expression of *sqnR* at high cell density. We further discovered that magnesium supplementation could alleviate this density dependence, although its action was independent of PhoP. Finally, we revealed that the nitrogen-responsive regulators GlnR and AfsQ1 could relieve the repression exerted by Lsr2 and PhoP. Intriguingly, we found that this low density-dependent production of saquayamycin was not unique to WAC07094; saquayamycin production by another wild isolate also exhibited low-density activation, suggesting that this spatial control may serve an important ecological function in their native environments.

**IMPORTANCE:**

*Streptomyces* specialized metabolic gene clusters are subject to complex regulation, and their products are frequently not observed under standard laboratory growth conditions. For the wild *Streptomyces* isolate WAC07094, production of the angucycline-family compound saquayamycin is subject to a unique constellation of control factors. Notably, it is produced primarily at low cell density, in contrast to the high cell density production typical of most antibiotics. This unusual density dependence is conserved in other saquayamycin producers and is driven by the pathway-specific regulator SqnR, whose expression is influenced by both nutritional and genetic elements. Collectively, this work provides new insights into an intricate regulatory system governing antibiotic production and indicates there may be benefits to including low-density cultures in antibiotic screening platforms.

## INTRODUCTION

Microbial specialized metabolites are important sources and inspirations for antibiotic discovery and development (1–3). *Streptomyces* bacteria and their actinobacterial relatives are major contributors to the antibiotics used in clinical and agricultural settings. These microbes also produce a wide array of biologically active molecules having anti-fungal, anti-cancer, immunosuppressant, and herbicide utility. The synthesis of any specialized metabolite is a complex and highly regulated process, and the production of these compounds is thought to confer their producers with a competitive advantage in their natural environment. Consequently, these molecules are often not produced at significant levels under conventional laboratory growth conditions.

Antibiotic production and specialized metabolism, more broadly, are integrated into the *Streptomyces* developmental cycle, where they are temporally linked with the onset of aerial (reproductive) growth (4). The transition from vegetative hyphal growth to the raising of aerial hyphae, formation of reproductive spores, and initiation of specialized metabolism is, in turn, linked to conditions of nutrient depletion, including low levels of carbon, nitrogen, and phosphate (5–7). Quorum sensing has also been tied to both development and antibiotic control (8), implicating a role for cell signaling and cell density in regulating specialized metabolism. Most characterized specialized metabolite biosynthetic clusters are subject to multilevel regulation (9). These clusters often encode dedicated cluster-situated regulators, many of which are transcriptional activators. These regulatory genes, in turn, are subject to control by multiple globally acting transcription factors, ranging from nutrient-sensing regulators like DasR (*N*-acetylglucosamine) (7), GlnR (nitrogen) (10), and PhoRP (phosphate) (11), through to nucleoid-associated proteins like Lsr2 (12). There are further instances when binding sites for these different regulators overlap (13, 14); this adds to the regulatory complexity governing specialized metabolism and provides opportunities for both distinct regulatory inputs and interplay between different regulators.

The nucleoid-associated protein Lsr2 is conserved throughout the actinobacteria and is functionally similar to the well-studied H-NS protein from *Escherichia coli* in serving as a silencer of horizontally acquired genes (15). In the streptomycetes, Lsr2 also represses the expression of many genes involved in specialized metabolism (12). This repression can be overcome by sequestering the native protein through overexpression of a dominant negative variant that is dimerization/oligomerization proficient but DNA binding defective (12). Constitutively expressing this “Lsr2 knockdown” construct has proven useful in stimulating the production of new metabolites by wild *Streptomyces* sp., presumably by relieving the biosynthetic cluster silencing exerted by the native protein (12).

We introduced this Lsr2 knockdown construct into a suite of wild *Streptomyces* isolates and identified one recipient that had enhanced antibiotic production relative to its parent strain. Unusually, its antibiotic production was most pronounced when cells were growing at low density; there was no activity seen for high-density cells, which is the converse of what is usually seen for antibiotic synthesis (16). We first determined that the antibiotic with these intriguing production characteristics was saquayamycin, a molecule that belongs to the angucycline family of natural products (17, 18). To better understand the mechanistic basis for the low cell-density production of saquayamycin, we probed the effects of both environmental factors (diverse nutrients), and genetic regulators. We found that magnesium salts relieved the density dependence and significantly enhanced saquayamycin production yields. From a regulatory perspective, beyond the stimulatory effects observed for Lsr2 knockdown, we further identified the two-component system PhoP/PhoR as a key regulator of saquayamycin. Notably, we determined that the low cell-density dependence of saquayamycin production was conserved in other saquayamycin producers and was subject to the same regulatory and nutritional controls in these other species.

## RESULTS

### Lsr2 knockdown promotes new antibiotic production for WAC07094 on agar medium

**Fig 1:**
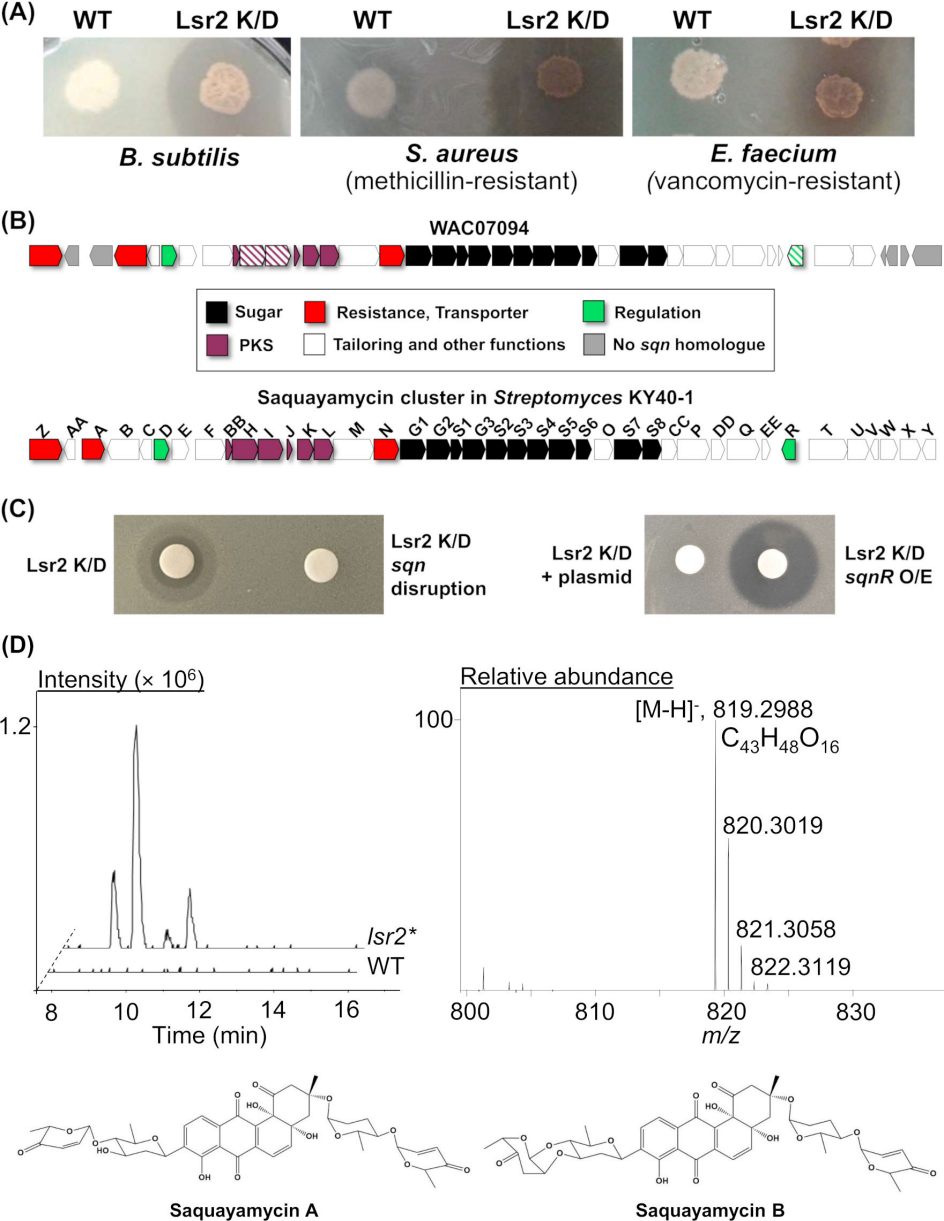
Saquayamycin production by *Streptomyces* sp. WAC07094. (**A**) Antibiotic bioassay in which wild type (WT) WAC07094 and Lsr2 knockdown (K/D) strains (overexpressing a dominant negative variant of *lsr2*) were spotted to Bennett’s medium, grown for 3 days, and then overlaid with the sensitive indicator strains *Bacillus subtilis* (left), methicillin-resistant *Staphylococcus aureus* (middle), or vancomycin-resistant *Enterococcus faecium* (right). (**B**) Schematic diagram of the saquayamycin biosynthetic cluster in *Streptomyces* KY40-1 (bottom), compared with the equivalent cluster from WAC07094 (top). Genes from the WAC07094 strain studied here are indicated with hashed lines. (**C**) Antibiotic bioassay using *B. subtilis* as the indicator strain, together with crude extracts spotted to filter disks, from the *sqnHI* disruption mutant and the *sqnR* overexpression strains (both carrying the Lsr2 knockdown construct) grown for 3 days on Bennett’s medium, compared with their relevant controls. (**D**) Liquid chromatography-mass spectrometry analysis of crude extracts prepared from WAC07094 grown for 3 days on Bennett’s agar (agar + biomass). (Left) Chromatograms of [M-H]^−^ ions extracted at *m*/*z* of 819; (right) mass spectra of the peak at *m/z* of 819 and its assigned chemical formula; (bottom) structures of saquayamycin A and B.

In an effort to activate silent biosynthetic gene clusters, we introduced our Lsr2 knockdown plasmid into a suite of wild *Streptomyces* isolates and screened these genetically modified strains for new antibiotic production. We found that introducing this construct into *Streptomyces* WAC07094 [Wright Actinomycete Collection (19)] led to new pigmentation and strong antibiotic activity against Gram-positive bacteria, including methicillin-resistant *Staphylococcus aureus* and vancomycin-resistant enterococci, in contrast to its plasmid-free or empty plasmid-containing parental strains, which were unpigmented and had either no or significantly reduced levels of growth inhibition (Fig. 1A; Fig. S1).

To identify the specialized metabolite (and its associated biosynthetic gene cluster) responsible for this new bioactivity, the WAC07094 genome was sequenced. The final genome assembly consisted of four contigs having a total length of 9,599,672 bp (accession number JAVMJZ000000000), including 18 biosynthetic gene clusters predicted using antiSMASH v.5.0 (20) (Table S1). In analyzing the annotated genome, we found that WAC07094 was unusual in encoding three *lsr2*-like genes, with two sharing strong similarity with the *lsr2* and *lsrL* genes encoded by other streptomycetes. The third gene encoded a protein that was more similar to Lsr2 than LsrL and was dubbed “LsrS,” for Lsr2 similar (Fig. S2). The promoter region of *lsrS* was highly similar (95% identity over 240 nt with no gaps) to that of the *lsr2* promoter, but the flanking genes differed from those adjacent to the canonical *lsr2* and included a putative transposase-encoding gene, suggesting that *lsrS* may have arisen through a genome duplication and transposition event.

We knew from previous work that Lsr2 binds AT-rich regions (12). Consequently, we assessed the relative AT content associated with each of the 18 predicted biosynthetic clusters and found that several clusters had AT-rich regions that could be targeted by Lsr2 (Table S1). As Lsr2 typically represses gene expression, we predicted that Lsr2 knockdown would be associated with increased expression of at least one of these biosynthetic clusters, leading to the observed antibiotic activity. To identify this cluster, RNA was isolated from wild type and Lsr2 knockdown strains after 3 days (when anti-bacterial activity was observed), and these samples were subjected to RNA sequencing. Comparing the levels of sequencing reads (accession number PRJNA1009436) between strains revealed that a polyketide synthase-encoding gene cluster with an extended AT-rich region was upregulated in the Lsr2 knockdown strain (Fig. S3). This cluster bore 85% similarity to the previously characterized saquayamycin biosynthetic gene cluster (MIBiG accession number BGC0001769) (21) but lacked the *sqnAA*, *sqnA*, and *sqnV-Y* genes (Table S2; Fig. 1B). To confirm that this cluster was indeed responsible for the new antibiotic activity seen for WAC07094, we created insertion mutations in key polyketide synthase genes within the cluster (*sqnH* and *sqnI*; Fig. 1B; Table S2) and, in parallel, overexpressed the predicted regulatory gene *sqnR*. The mutant strains exhibited a complete loss of antibiotic activity, while overexpressing the SqnR regulator led to enhanced antibiotic activity (Fig. 1C).

In parallel, we analyzed the metabolic profiles of crude extracts from wild type and Lsr2 knockdown strains using liquid chromatography-mass spectrometry (LC-MS). We found saquayamycin isomers A and B (with M-H ions at *m*/*z* 819.29 with a formula of C_43_H_48_O_16_) were readily detectable in the knockdown strain (Fig. 1D).

### Cell density impacts saquayamycin production and activity

**Fig 2:**
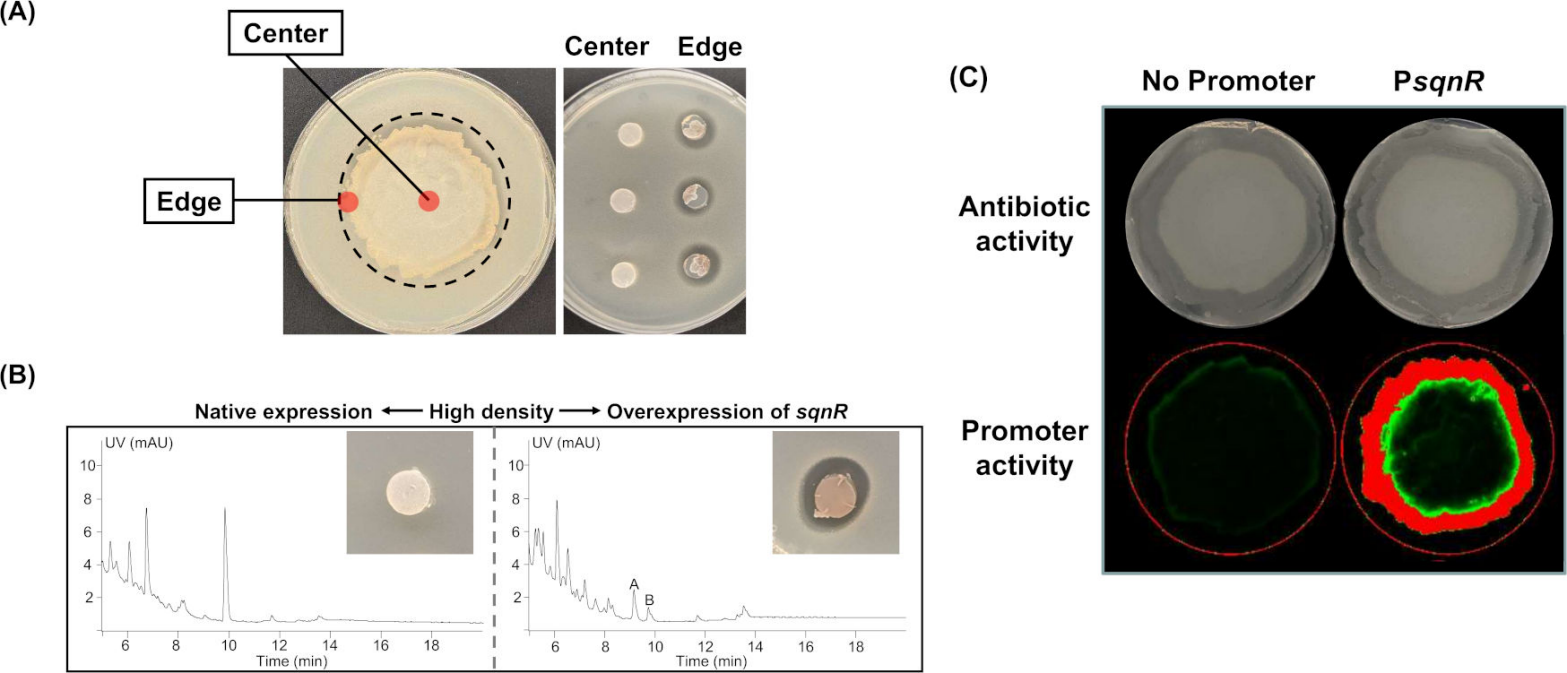
Saquayamycin activity and *sqnR* expression are density dependent. (**A**) (Left) Agar plugs were excised from the center and edge of a confluent lawn of the WAC07094 Lsr2 knockdown strain; (right) bioassays for antibiotic production, conducted by removing agar plugs (from the colony center or edge, as indicated on the left) of the Lsr2 knockdown strain, placing them onto *B. subtilis*-containing medium and incubating the plates overnight at 37°C. (**B**) Chromatograms (UV) of crude extracts prepared from Bennett’s agar on which the Lsr2 knockdown strain (left) and same strain carrying the *sqnR* overexpressing construct (right) had been grown as a lawn for 3 days. Peaks of saquayamycin A and B are indicated with the A and B labels, respectively. (Inset) Anti-*Bacillus* activity exerted by corresponding wild type (left) and *sqnR*-overexpressing (right) strains, where their associated agar plugs were taken from the center of a confluent lawn (high density). (**C**) (Top) Antibiotic bioassay of the Lsr2 knockdown strain carrying either the promoter-less *gfp* (left) or *sqnR* promoter-driven *gfp* construct, against *B. subtilis*. Zones of clearing indicate antibiotic activity; (bottom) promoter activity is seen as green fluorescence produced by the same *Streptomyces* strains, where red signals represent saturated fluorescence intensities (high promoter activity).

When conducting activity assays for the saquayamycin producer strain, we were surprised to observe that antibiotic activity was associated only with agar plugs taken from the edge of a lawn of WAC07094 and not with plugs taken from the center. This suggested that saquayamycin production might be subject to spatial control, with antibiotic production only being activated at the colony edge and/or suppression only occurring at the colony center (Fig. 2A). To assess whether this was a cell density-dependent phenomenon, we plated a spore dilution series and evaluated the resulting growth (including both individual colonies and more confluent areas) for antibiotic production using an activity bioassay. We found that activity against *Bacillus subtilis* was inversely proportional to spore density, with increasing colony numbers and greater confluence being associated with reduced antibiotic production (Fig. S4).

To confirm that the enhanced activity observed during low-density growth was due to saquayamycin, metabolites were extracted from agar medium associated with either high-density or low-density cells. Saquayamycins were detected exclusively in extracts from low-density growth areas (Fig. S4), and we found this density-dependent effect was alleviated when the pathway-specific regulator *sqnR* was overexpressed (Fig. 2B), suggesting that the spatial control of antibiotic production may be mediated through *sqnR*. To test this hypothesis, we created a transcriptional reporter fusion using the *sqnR* promoter and a promoterless green fluorescent protein (GFP)-encoding gene. The resulting construct, in parallel with the associated promoter-less control, were introduced into the Lsr2 knockdown strain where they integrated into the chromosome at a heterologous site. We followed the expression of the P*_sqnR_-gfp* reporter during growth as a lawn and found that GFP expression was observed only at the lawn periphery and was directly correlated with the sites of antibiotic activity (Fig. 2C).

### High cell-density repression of saquayamycin is alleviated by the addition of magnesium

**Fig 3:**
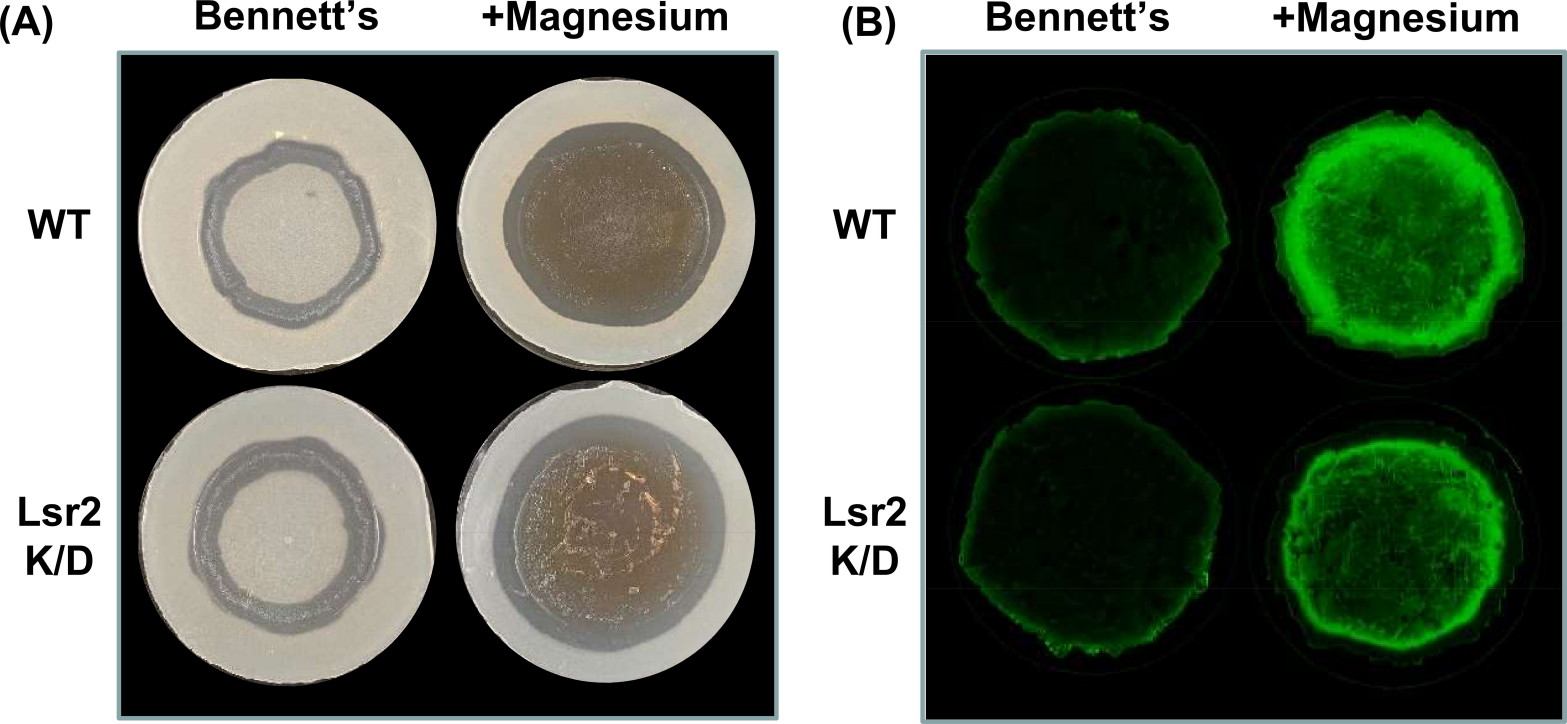
Density-dependent repression of saquayamycin production is alleviated by magnesium supplementation. (**A**) Antibiotic bioassay of wild type (WT) and Lsr2 knockdown (K/D) strains (carrying the *sqnR* transcriptional reporter) grown for 3 days on Bennett’s medium in the absence (left) or presence (right) of magnesium supplementation, where the resulting lawns of the two strains were overlaid with soft agar infused with *B. subtilis*. Zones of clearing indicate antibiotic activity. (**B**) *sqnR* promoter activity of the strains shown in panel **A**, as measured by green fluorescence of different biological replicates.

To begin to understand the basis for the density/spatial specificity of *sqnR* expression and, correspondingly, saquayamycin activity, we first considered the effect of nutrients. We tested the impact of various carbon and nitrogen sources, as well as trace metals and salts, on saquayamycin production. We found that magnesium supplementation alleviated the density-dependent suppression of saquayamycin production, with antibiotic production now being observed across the entire colony area (Fig. 3A). This effect seemed specific to magnesium, as neither zinc nor manganese had an equivalent effect (Fig. S5). We further tested whether the magnesium-dependent antibiotic stimulatory effect was mediated through *sqnR* using our fluorescent reporter constructs. We found that magnesium supplementation led to *sqnR* expression throughout the colony, again correlating with antibiotic activity (Fig. 3B). These results suggested that *sqnR* expression and, correspondingly, saquayamycin production could be stimulated by magnesium in a density-independent manner.

### Magnesium and PhoRP independently control saquayamycin biosynthesis

In addition to the effect of nutrients from the environment, we considered the possibility that saquayamycin production might be also governed by quorum sensing. We searched the genome of WAC07094 for genes that may direct the synthesis of extracellular signaling molecules, including butyrolactone and butanolide synthases (commonly used by streptomycetes to synthesize gamma-butyrolactone/butanolide signaling molecules), as well as small peptides like those employed in quorum sensing by other Gram-positive bacteria. Unexpectedly, no strong candidates for genes encoding signaling peptides or butyrolactone/butanolide synthases were found in the genome.

**Fig 4:**
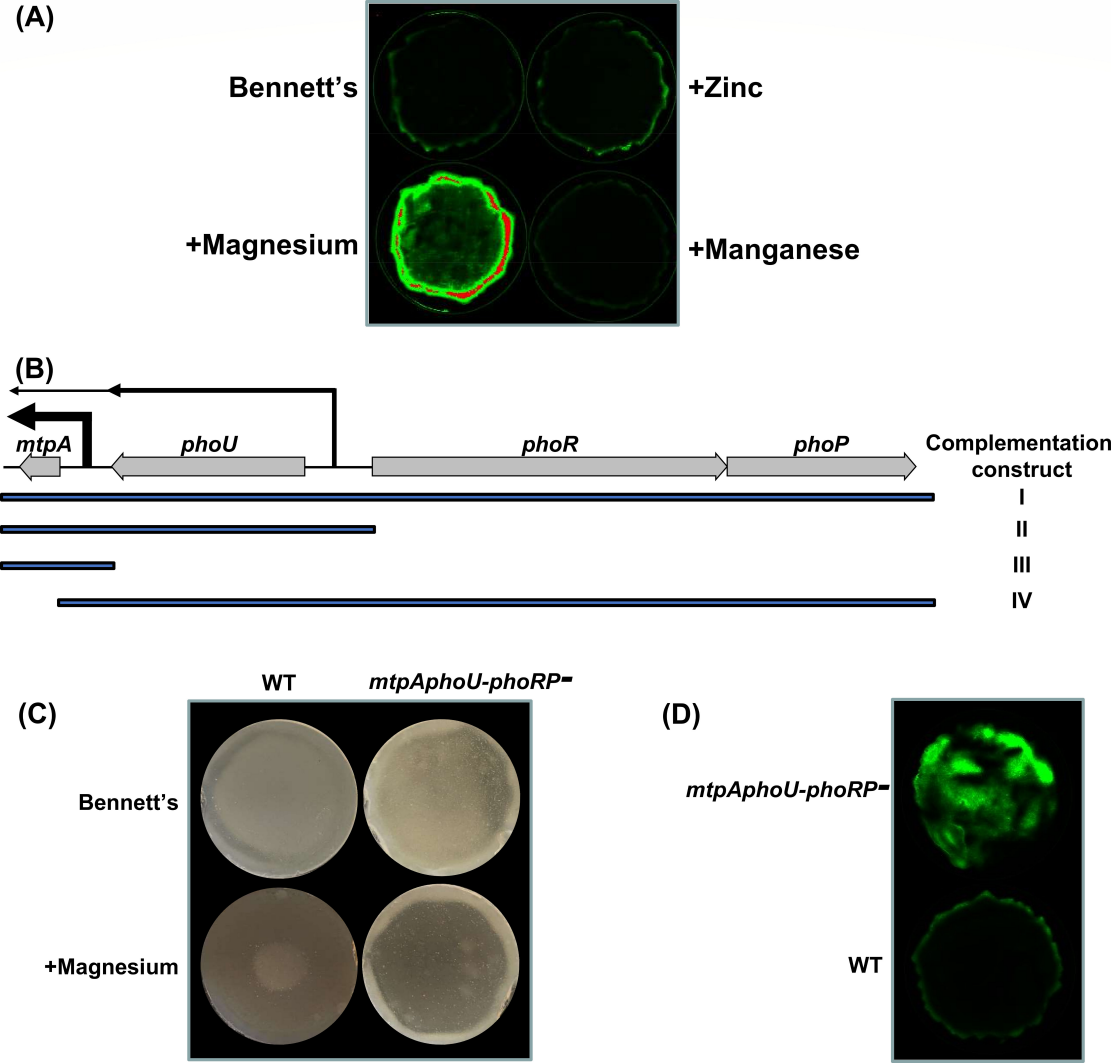
The density-dependent expression of *mtpA* and the saquayamycin cluster is affected by magnesium and PhoRP. (**A**) Promoter activity of *mtpA* in wild-type WAC07094 after 3 days’ growth on Bennett’s agar with or without magnesium chloride, manganese chloride, or zinc chloride supplementation. (**B**) Genetic organization of the *mtpA*, *phoU*, *phoR*, and *phoP* locus. Transcription start sites are indicated by vertical lines, with the line widths (and associated horizontal arrows) approximating relative transcript abundance. Beneath the genes are four lines, indicating the sequence included in each of four complementation constructs (labeled I–IV on the right). (**C**) Antibiotic activity as detected using *B. subtilis* infused agar, overlaying lawns of wild type (WT) or mutant strains grown for 3 days on Bennett’s agar, with or without additional magnesium supplementation. (**D**) Promoter activity of *sqnR* after 3 days of growth, as indicated by the relative fluorescence within the lawn of the wild type and *phoRP* locus mutant.

When searching for small peptide-encoding genes, however, we did identify one termed *mtpA* that was predicted to encode a small metal-binding protein (22) and wondered whether there may be a connection between the resulting MtpA protein, magnesium, and saquayamycin production. To localize *mtpA* expression, we constructed a transcriptional fusion between the *mtpA* promoter and the promoterless *gfp* reporter gene and introduced the resulting construct into the wild-type strain, where it integrated into the chromosome at a heterologous site. We found that, like *sqnR*, *mtpA* expression was enhanced at the colony periphery and was increased throughout the colony following magnesium (but not manganese or zinc) supplementation (Fig. 4A).

We noted that *mtpA* was located immediately downstream of *phoU* (Fig. 4B). Our RNA-seq data suggested that *mtpA* was expressed primarily from its own promoter but that read-through transcription from *phoU* also contributed to its expression (Fig. 4B). These two genes, in turn, were divergently oriented from the *phoRP* two-component system-encoding genes (Fig. 4B). To determine whether there were connections between any of these genes/proteins and saquayamycin production, the four-gene (*mtpAphoU-phoRP*) locus was deleted in the wild-type background, and both *sqnR* expression and antibiotic activity were monitored. Interestingly, the mutant phenotype was reminiscent of colonies growing with magnesium supplementation, although not quite as robust (inhibition was ~75% of that seen with magnesium supplementation): anti-bacterial activity and *sqnR* expression were now detected throughout the colony, not just at the colony edges (Fig. 4C and D, respectively). This suggested that the density-dependent saquayamycin production phenotype could be facilitated at least in part by one or more of the genes in the *mtpAphoU-phoRP* locus. We also observed that the loss of *mtpA* (as part of this four gene locus) did not impact the magnesium-mediated stimulation of saquayamycin production (Fig. 4C), suggesting that this effect did not require MtpA activity.

To determine which of the gene products within this locus was responsible for the density-dependent saquayamycin biosynthesis phenotype, we constructed a suite of complementation constructs (Fig. 4B) which were introduced into the *mtpAphoU*-*phoRP* mutant strain. We first confirmed that complementation with the four-gene locus restored the wild-type phenotype, with antibiotic production only being observed at the colony periphery (Table 1). Complementation with *phoRP*, together with *phoU* (lacking *mtpA*), had an identical effect, with saquayamycin activity being restricted to the colony periphery (Table 1). This suggested that MtpA was not responsible for the spatial localization of saquayamycin production. In contrast, when *mtpA* and *phoU* were reintroduced (without *phoRP*), colony-wide antibiotic production was retained. This indicated that the spatial nature of saquayamycin production was likely due to PhoRP-mediated repression of *sqnR* expression in high-cell density areas.

**TABLE 1:** Antibiotic activity phenotype of the *mtpAphoU*-*phoRP* complementation strains

Strain	Genotype^*a*^				Phenotype (inhibition of *B. subtilis*)
	*mtpA*	*phoU*	*phoR*	*phoP*	
Wild-type	+	+	+	+	Weak inhibition at the colony periphery
*mtpAphoU-phoRP*	−	−	−	−	Colony-wide inhibition
Complemented I	+	+	+	+	Weak inhibition at the colony periphery
Complemented II	+	+	−	−	Colony-wide inhibition
Complemented III	+	−	−	−	Colony-wide inhibition
Complemented IV	−	+	+	+	Weak inhibition at the colony periphery

^*a*^ (+) indicates that the strain/complementation construct contains this gene; (−) indicates that the strain/complementation construct does not contain this gene.

### Saquayamycin production is impacted by multiple regulators

**Fig 5:**
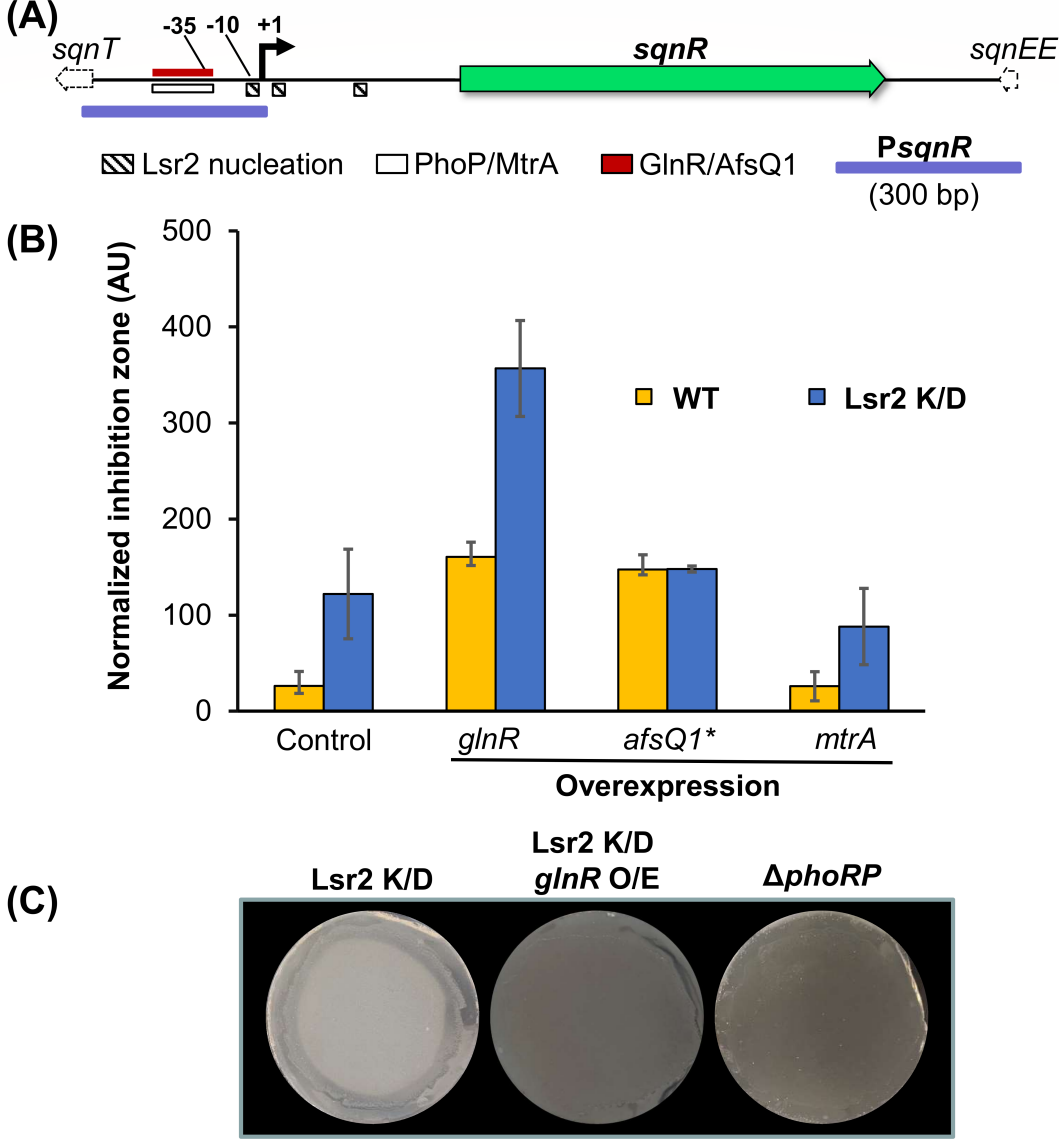
GlnR positively controls saquayamycin production. (**A**) Schematic of the *sqnR* promoter region, including the transcription start site (+1), the upstream promoter (−10 and −35), and the predicted binding sites for Lsr2, PhoP, MtrA, GlnR, and AfsQ1. The promoter region used for the reporter constructs is indicated in purple. (**B**) Antibiotic bioassay using WAC07094 wild type (WT) or Lsr2 knockdown (K/D) strains carrying either the empty plasmid pIJ10257 (control), or *glnR* or *mtrA* under the highly active ermE* promoter, or afsQ1* under the inducible *tipA* promoter. *B. subtilis* was used as the sensitive indicator strain, and growth inhibition zones were quantified and normalized to the corresponding *Streptomyces* growth area. Error bars indicate standard deviation (*n* = 4). (**C**) The effect of *phoRP* deletion relative to Lsr2 knockdown, and Lsr2 knockdown together with *glnR* overexpression, were assessed using *B. subtilis*-infused agar overlays. The darker coloration associated with the two rightmost strains indicates full growth inhibition, in contrast to inhibition only at the periphery seen for the leftmost plate.

The Pho system controls phosphate transport, and the PhoRP system is activated in response to low phosphate levels (11). This results in phosphorylation of the PhoP response regulator, which enables its binding to the promoters of target regulon members at well-defined Pho-box sites (23, 24), altering the expression of the associated genes. Given the negative effect of PhoRP on *sqnR* expression—and saquayamycin production—in high-density colony areas (Fig. 4C and D), we examined the *sqnR* promoter for possible PhoP-binding sites. We identified a candidate PhoP-box located 28 nt upstream of the predicted transcription start site of *sqnR* within the likely promoter region (Fig. 5A). An equivalent box [direct repeat units GG/TTCAYYYRC/GG (23)] was also found upstream of *phoU.* Beyond PhoP, the binding sites of many other global regulators of antibiotic production have been defined, and a number of these share core recognition sequences with PhoP, including GlnR and the two component regulators MtrA and AfsQ1 (Fig. 5A) (9), and thus may also impact *sqnR* expression and saquayamycin production. We overexpressed each regulator-encoding gene [for *afsQ1*, we overexpressed a mutant version expressing a phosphomimic variant (25)] to determine whether any of these impacted saquayamycin production in either wild-type or Lsr2-knockdown backgrounds. As expected, enhanced anti-bacterial activities were observed for all Lsr2 knockdown-carrying constructs (Fig. 5B). We found that overexpressing *mtrA* (together with its associated kinase-encoding *mtrB*) reduced saquayamycin production, while overexpressing *glnR* and afsQ1* [from constitutive (ermE*) and inducible (*tipA*) promoters, respectively] enhanced saquayamycin production in both wild-type and Lsr2 knockdown backgrounds (Fig. 5B and C). In the case of afsQ1*, its effects were most pronounced in the wild-type background, with Lsr2 knockdown having no additive effect in an afsQ1* overexpressing strain (Fig. 5B). This suggested that AfsQ1 may function to counteract the repressive function of Lsr2. In contrast, overexpressing *glnR* in the wild-type background was at least as effective as Lsr2 knockdown in enhancing saquayamycin production, while in the Lsr2 knockdown background, the effects were additive and mirrored the effect of *phoRP* deletion. This collectively suggested that PhoP and Lsr2 were the predominant repressors of saquayamycin production, while GlnR and AfsQ1 contributed to saquayamycin activation.

### Saquayamycin biosynthesis is activated in low-density inocula liquid cultures

**Fig 6:**
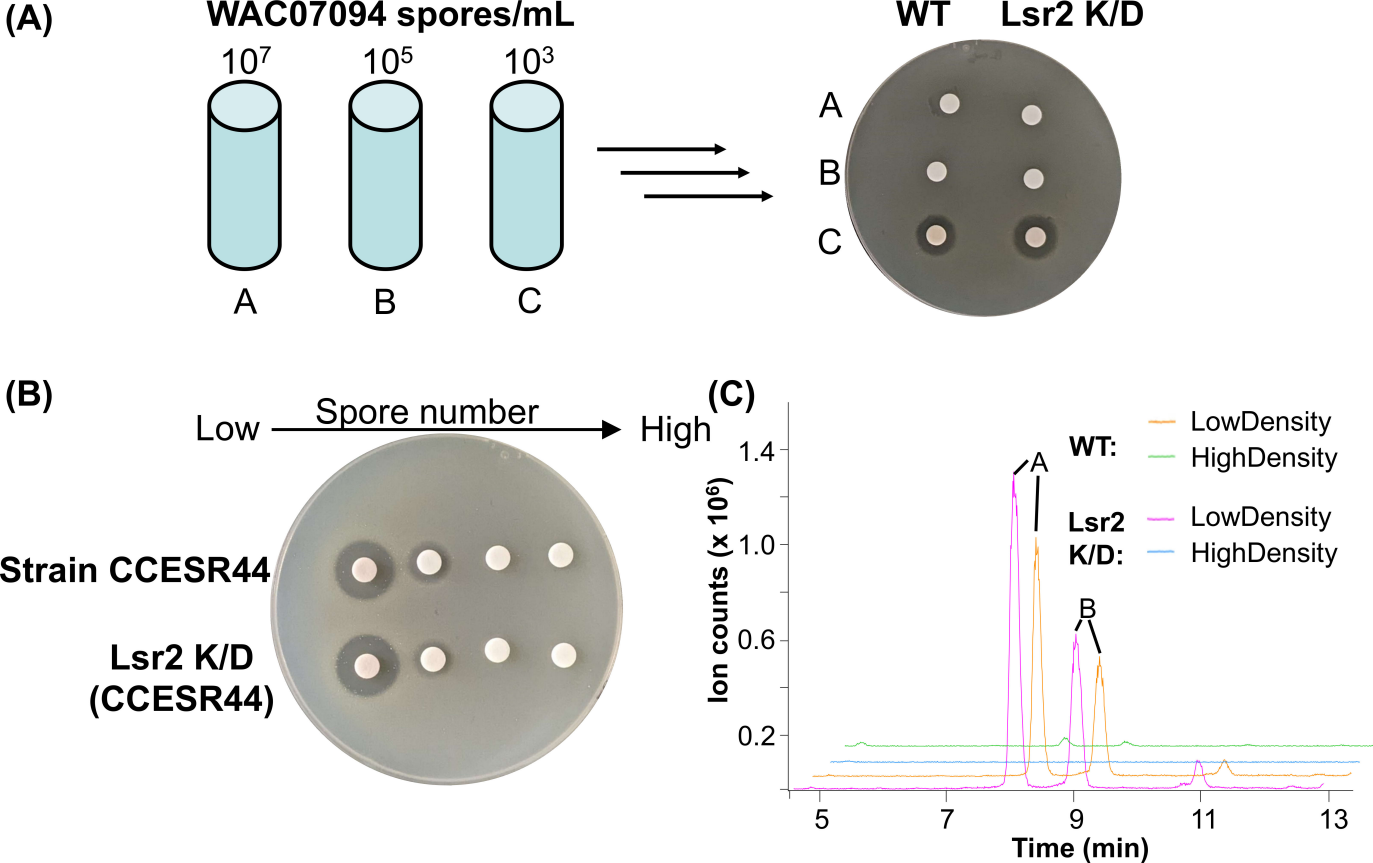
The density dependence of saquayamycin production is conserved across diverse streptomycetes. (**A**) Antibiotic bioassays were conducted using *B. subtilis* as the sensitive indicator organism, together with extracts of wild type (WT) and Lsr2 knockdown (K/D) WAC07094 cultures inoculated from low-density (10^3^/mL), mid-density (10^5^/mL) and high-density (10^7^ /mL) spore preparations and grown for 4 days in Bennett’s liquid medium. (**B**) Experiments equivalent to that in panel **A** were conducted for *Streptomyces* isolate CCESR44 wild type and Lsr2 knockdown strains, including only an additional low-density inoculum of 100–200 spores/mL. (**C**) Extracted ion chromatograms of [M-H]^−^ (at *m*/*z* 819.3) from metabolite profiles for low- and high-cell density inoculated cultures from panel** B**.

Given the intriguing density-dependent production of saquayamycin during growth on solid agar medium, we wondered whether an equivalent density dependence might also be observed for liquid-grown cultures. We tested the antibiotic production activity of both wild-type and Lsr2 knockdown strains using high- and low-concentration spores as inocula in Bennett’s liquid medium. We found that both strains produced saquayamycin exclusively in cultures inoculated with low numbers of spores (Fig. 6A). We tested whether magnesium supplementation or *phoRP* deletion could enhance saquayamycin production when starting with a higher-spore inoculum. We found that unlike on solid medium, magnesium failed to stimulate saquayamycin production during “higher-density” (higher spore inoculum) growth. In contrast, *phoRP* mutants produced high levels of saquayamycin under the same growth conditions (Fig. S6).

### Control of saquayamycin production is conserved between different producer strains

To investigate whether the low-cell density production phenotype was shared more broadly among saquayamycin producers, we searched for strains with this biosynthetic cluster using the National Center for Biotechnology Information and Joint Genome Institute databases (26, 27) and found a candidate in *Streptomyces* sp. 3212.3 (accession number QTTM01000001.1), which has subsequently been termed isolate CCESR44 (L. Kinkel, personal communication). We compared its annotated saquayamycin biosynthetic cluster to that of WAC07094 and found only minor differences at the cluster boundaries (Fig. S7A). Phylogenetic comparisons between these strains and several reference genomes (*de novo* mode in https://automlst.ziemertlab.com/index) (28) were conducted using ~50 select single-copy conserved genes (Fig. S7B). These analyses suggested that strains WAC07094 and CCESR44 were unique but shared 99% identity between 16S rRNA sequences (comparing all six copies of the genes); they grouped closely with each other and with *Streptomyces* sp. NRRL F-5122 and *Streptomyces nodosus* ATCC 14899. Strains WAC07094 and CCESR44 were also observed to have different colony morphologies (Fig. S7C).

We tested the effect of cell density on saquayamycin production by CCESR44. Interestingly, this strain was also observed to exclusively produce saquayamycin when grown using a low-density inoculum in liquid culture (Fig. 6B). Introducing our Lsr2 knockdown construct into this strain further increased antibiotic activity (Fig. 6B and C). We confirmed that this activity was due to saquayamycin using chemical analyses, alongside genetic tests, where creating insertion mutations in the polyketide synthase genes *sqnH* and *sqnI* abolished antibiotic activity (Fig. S7D; Fig. 6C). We further tested the effects of magnesium supplementation during growth on solid medium, and as for WAC07094, we observed antibiotic production throughout the entire colony, irrespective of cell density (Fig. S8). These observations collectively suggest that the spatial, genetic, and nutritional controls governing saquayamycin production are conserved across disparate species.

## DISCUSSION

The conventional view of specialized metabolism in the streptomycetes is that it occurs in mature, high-density cultures. Our work here suggests that there are conserved exceptions to this rule, where antibiotic production can instead be confined to low-cell density areas. In bacteria, cell density can impact a wide range of processes (16, 29, 30). One of the best-studied density-dependent behaviors involves quorum sensing, which is mediated predominantly by butyrolactone or butanolide signaling molecules in *Streptomyces* spp. (31). While there were no obvious quorum signaling systems encoded in the genome of WAC07094, our work suggests that this species effectively exerts density-dependent control of the saquayamycin biosynthetic genes via the cluster-situated regulator *sqnR*.

**Fig 7:**
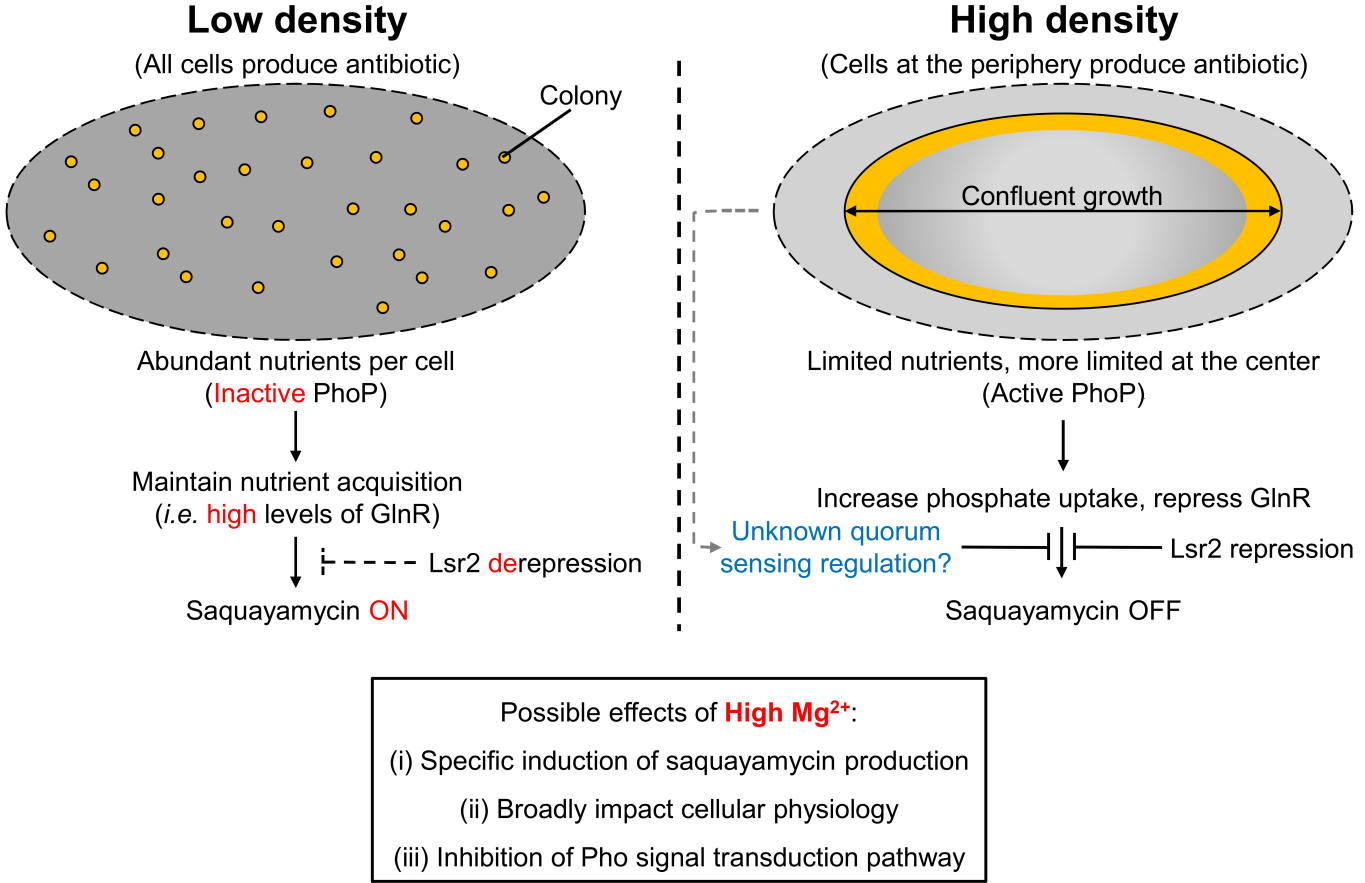
Proposed model of the regulatory and nutritional inputs impacting saquayamycin production, both highlighting known features of the system and acknowledging the possibility of additional (as yet unknown) contributing systems.

We found that there were multiple factors that influenced the density-dependent production of saquayamycin (Fig. 7). Magnesium was a particularly potent activator of *sqnR* expression during solid culture growth. We were not able to distinguish whether this was the result of specific induction of *sqnR* transcription or if it reflected a broader impact on cellular physiology; however, it is worth noting that the saquayamycin-enhancing effect of magnesium was conserved in both *Streptomyces* spp. WAC07094 and CCESR44. In other bacteria, magnesium has been implicated in everything from DNA replication (32) and gene regulation (33–35), through to protein folding and stability (36). It has also been associated with alternative oligomeric assemblages for the nucleoid-associated protein H-NS (37), which is functionally equivalent to the Lsr2 protein in the actinobacteria. Whether magnesium impacts Lsr2 behavior is currently unknown; it is tantalizing to speculate that it may help to alleviate the Lsr2-mediated repression observed for the saquayamycin biosynthetic cluster.

The effects of magnesium on gene regulation have been best studied in enterobacteria (*Salmonella*, *Yersinia*, and *Escherichia*), where magnesium levels modulate the activity of the PhoP/PhoQ two-component regulatory system (38). The streptomycetes do not have an equivalent system; instead, they and their Gram-positive relatives possess a PhoP/PhoR system, which is analogous to PhoB/PhoR in the enteric bacteria. In *Salmonella*, increased magnesium levels also negatively affect RNA polymerase occupancy and mRNA transcript levels of PhoB regulon members, including genes for the phosphate transport system (*pstSCAB*), and *phoBR* itself (39). While the effect of magnesium on PhoP/PhoR activity in the streptomycetes is unknown, a similar effect could lead to reduced PhoP levels. Notably, deletion of *phoRP* led to constitutive *sqnR* production and enhanced saquayamycin production, similar to the phenotype observed following magnesium supplementation. This suggested that PhoP functioned to repress *sqnR* expression in areas of confluence/high cell density and was further supported by the presence of a Pho box upstream of the *sqnR* transcription start site. We cannot, however, exclude the possibility that PhoP controls the synthesis of a novel quorum (or anti-quorum)-sensing compound that impacts *sqnR* expression. There is precedent for connections between phosphate regulation and quorum sensing in *Pseudomonas aeruginosa*, where production of the integrated quorum sensing (IQS) signaling molecule is governed by PhoB, the PhoP equivalent (40).

In the absence of PhoP repression, we propose that transcription activator(s) can access the *sqnR* promoter and recruit RNA polymerase to help counter the downstream Lsr2 silencing of the saquayamycin gene cluster (41). A likely candidate for this role is the nitrogen-responsive GlnR regulator. There is a predicted GlnR-binding site upstream of the *sqnR* promoter, and overexpression of GlnR led to increased saquayamycin production. Furthermore, there is an intriguing reciprocal relationship shared by GlnR and PhoP in many streptomycetes (9) that mirrors the situation with saquayamycin production. PhoP can directly repress GlnR, and thus, as PhoP levels drop, GlnR levels would rise. In WAC07094, this could allow GlnR to both activate *sqnR* transcription and promote the RNA polymerase-mediated removal of Lsr2 from the chromosome, alleviating its repressive effects. AfsQ1, which shares the same DNA target sequence with GlnR, can also promote saquayamycin production, and this appears to be largely through alleviation of Lsr2 repression.

The ecological roles of most natural products are not well understood. Saquayamycins are pigmented, glycosylated natural products, and they have growth-inhibitory activity against both Gram-positive bacteria and eukaryotes. Indeed, the activity of the saquayamycins and other angucycline family members have been best studied for their anti-tumor and enzymatic inhibitory properties (42). Paradoxically, angucyclines have been reported to be produced by endophytes, microbes that live inside plants and sponges (43, 44). We speculate that the producers of these compounds, and in the case of endophytes, also their hosts, must benefit from the synthesis of these toxic compounds, possibly as a result of their anti-fungal properties. In the case of free-living bacteria like WAC07094 and CCESR44, the production of saquayamycin at the colony edge may function to stave off competing microbes and secure sufficient nutrients to promote continued growth and/or reduce the likelihood of predation by other soil-dwelling organisms. It is notable that saquayamycins are synthesized in many forms (here we identified saquayamycins A and B). Whether different forms have distinct functions or properties remains to be determined.

Microbes are an extraordinary source of biologically active molecules. Understanding the interplay between gene regulation, physiology, and metabolism is important to maximize the biosynthetic capacity of these organisms and to exploit the functional landscape of new molecules. Beyond demonstrating the power of our Lsr2 knockdown approach in stimulating saquayamycin production and uncovering the interrelated impacts of magnesium, PhoP, GlnR, and AfsQ1, our work here has also revealed the unexpected importance of low cell density on specialized metabolism. Whether low-cell density production of natural products is a rare or common phenomenon remains to be determined, but it is worth noting that the regulators involved in controlling saquayamycin production (Lsr2, PhoP, GlnR, and AfsQ1) are broadly conserved in the actinobacteria. This observation is, however, not without precedent: in 2011, a blue-pigmented molecule was found to be produced exclusively at lower initial cell densities by the plant pathogenic bacterium *Pantoea agglom*erans (45). These findings suggest there may be benefits to adding low-cell density cultures to natural product screening platforms.

## MATERIALS AND METHODS

### Bacterial strains, plasmids, and culture conditions

Bacterial strains, plasmids, and oligonucleotides used in this study are listed in supplemental Tables S3 and S4. *E. coli* DH5α was used for general subcloning and plasmid preparation (46). *E. coli* strains were grown at 37°C in lysogeny broth or on Difco nutrient agar. The non-methylating *E. coli* strain ET12567/pUZ8002 was used as a donor host for intergeneric conjugation (47). *Streptomyces* strains were grown at 30°C in Bennett’s liquid medium or on Bennett’s agar (per liter, 10-g glucose, 1-g yeast extract, 1-g beef extract, 2-g N-Z amine, without or with 15-g agar) (48) or cultured on International *Streptomyces* Project 4 agar (ISP4) (49) unless otherwise stated. Antibiotic selection was carried out using antibiotics at the following concentrations: apramycin (50 µg/mL), chloramphenicol (25 µg/mL), kanamycin (50 µg/mL), nalidixic acid (25 µg/mL), and hygromycin (50 µg/mL). The indicator strains used in the anti-microbial assays included *Bacillus subtilis*, methicillin-resistant *Staphylococcus aureus*, and vancomycin-resistant *Enterococcus faecium*. These strains were grown at 37°C in tryptic soy broth or on Difco nutrient agar.

### Whole-genome sequencing

Genomic DNA was prepared using a salting-out protocol (48), after which RNA was removed using an RNase A treatment. DNA quality and quantity were assessed using agarose gel electrophoresis and a Nanodrop spectrophotometer (Thermo Fisher). A draft genome sequence for *Streptomyces* sp. WAC07094 was obtained using both Illumina and PacBio sequencing. For Illumina sequencing, library preparation, sequencing, and read processing were conducted as described previously (50). For PacBio sequencing, the DNA library was prepared by the Farncombe Metagenomics Facility at McMaster University using the SMRTbell Template Prep Kit v.1.0 following PacBio’s Microbial Multiplexing protocol. Multiple libraries were pooled and size-selected using the BluePippin BLF75 kit with a high pass collection for fragments >7 kb. Sequencing was performed in the Farncombe Metagenomic sequencing facility using the Sequel Sequencing Kit v.3.0 reagents for a 20 hour-run on a SMRT Cell 1M. PacBio reads were assembled using the Flye assembler (51) in a computer cluster before being polished first with the built-in Flye system, followed by the gccp polisher (https://github.com/PacificBiosciences/gcpp). A “reference” sequence was aligned with PacBio raw reads using pbmm2 aligner (https://github.com/PacificBiosciences/pbmm2). The polished assembly was ultimately aligned with filtered and trimmed Illumina reads using minimap2 aligner (52). The overlapping sequences were used to create a consensus sequence between the Illumina and PacBio sequencing reads using Racon (53) to give a final assembly consisting of four contigs (8,469,185; 263,838; 536,999; and 329,400 bp). The contig sequences have been deposited in DDBJ/ENA/GenBank under accession number JAVMJZ000000000. The version described in this paper is version JAVMJZ010000000. Biosynthetic gene clusters were predicted using antiSMASH v.5.0 (20).

### Genetic manipulation of WAC07094

To modulate the activity of Lsr2 in WAC07094, a previously generated Lsr2 knockdown construct (pMC109) (12) was used. This plasmid was introduced into WAC07094 through intergeneric conjugation. About 10^8^ viable spores were used for conjugation on ISP4 supplemented with 20-mM MgCl_2_ after mixing with cells from 3 mL of *E. coli* ET12567/pUZ8002 overnight cultures. Representative apramycin-resistant exconjugants were selected for bioassays. As a control, the pIJ12551 (empty plasmid parent of the Lsr2 knockdown construct) was also introduced into WAC07094.

To disrupt the saquayamycin biosynthetic genes, a 2.3-kb homologous DNA fragment was cloned into a unique *Spe*I site of a non-replicative plasmid (pIJ10700) using T4 DNA ligase (Roche). The fragment was amplified using Q5 DNA polymerase (NEB) and primers DRsqnH-upSpeI/DRsqnI-downSpeI with a 61°C–68°C gradient annealing temperature (Bio-Rad S1000 thermocycler). The cloned fragment was confirmed by restriction analysis and sequencing using the universal M13-Reverse primer. Gel extraction and plasmid preparation kits were purchased from NEB and Invitrogen, respectively. The resulting disruption construct (pMC341) was introduced into the WAC07094 Lsr2 knockdown strain through conjugation and selection for hygromycin-resistant exconjugants.

To overexpress the transcriptional activator-encoding *sqnR*, the gene was cloned into the *Nde*I and *Xho*I sites, downstream of the constitutive ermE* promoter in the integrating plasmid pIJ10257. *sqnR* was amplified using primers EXsqnR-upNdeI/EXsqnR-downXhoI with a 62°C–67°C gradient annealing temperature. The resulting plasmid clones (pMC342) were sequenced using primer P*ermE* for verification before being mobilized into the WAC07094 Lsr2 knockdown strain. As above, the empty pIJ10257 was also introduced into the knockdown strain as a control.

### Antibiotic bioassays

To test their bioactivities, *Streptomyces* sp. WAC07094 strains or strain 3212.3 was inoculated onto Bennett’s agar plates in various ways, including (i) applying 5 µL of a spore suspension to yield a dense “spot” (e.g., Fig. 1); (ii) spreading spore suspensions (100 µL each) over an agar plate to obtain a lawn (e.g., Fig. 2); or (iii) spreading to obtain single colonies (e.g., Fig. S4A). In each instance, the resulting cells/colonies were grown for 3–5 days. The day before initiating the bioassay, indicator strains were inoculated and grown overnight, before being inoculated (0.1% vol/vol) into melted Difco nutrient agar. The resulting cell suspension was then poured into a petri plate. Subsequent antibiotic tests were conducted using either “agar plug” or “sandwich” assays. In the case of the former, agar plugs were removed from the lawn of *Streptomyces* growth and transferred onto the indicator plates. In the latter situation, the indicator-containing agar was removed from its petri dish once solidified and was overlaid atop the *Streptomyces* growth plate. For both plug and sandwich assays, the plates were incubated overnight at 37°C. Antibiotic effects on the indicator strain were observed as inhibition zones around the plugs or in/around the *Streptomyces* growth areas, and these were captured by camera (Pixel 3a). The inhibition zones were measured using ImageJ v.1.49 at 8-bit resolution (54) and were normalized against the growth area, which was also measured using the same software.

The antibiotic activity of crude extracts (the preparation for which is described below) was determined using 6-mm disk-diffusion assays on indicator plates. The indicator plates were prepared as described above, after which the 6-mm filter disks were placed on the plate, and 5–10 µL of crude extract was applied to the disks. Growth inhibition of the indicator strains was observed as zones of clearing around the disks after overnight incubation.

### Genome-wide transcriptome analysis using RNA-Seq

With a genome assembly in hand, we set out to assess the expression of biosynthetic clusters under conditions where bioactivity was observed for the Lsr2 knockdown strain. *Streptomyces* sp. WAC07094 was inoculated by spotting 5 µL of spore suspensions in sterile water (5,000–50,000 viable spores) on Bennett’s agar plates. After 3 days of growth, RNA was isolated from biomass scraped from the agar surface (~100 mg) using the Macherey-Nagel NucleoSpin RNA isolation kit with minor modifications. Cells were lysed using beads and chloroform prior to RNA purification using the spin column. To remove contaminating DNA, RNA samples were treated with TURBO DNAse (Ambion), and the resulting RNA preparations were then assessed for abundance and purity using a Nanodrop spectrophotometer, with RNA integrity being further assessed using agarose gel electrophoresis and an Agilent 2100 Bioanalyzer. To ensure there was no residual DNA in the RNA preparations, a housekeeping gene (16S rRNA gene) was targeted for amplification (35 cycles using primers listed in Table S4).

cDNA library construction and sequencing were performed by the Farncombe Metagenomics Facility at McMaster University using an Illumina MiSeq sequencer with a MiSeq v.3 reagent kit (2 × 75 bp paired-end configuration). Prior to library construction, ribosomal RNA was depleted using RiboZero (Illumina). Libraries were prepared using NEBNext Ultra II Directional RNA library prep kit (without size selection) and barcoded using NEBNext Multiplex Oligos for Illumina (96 Unique Dual Index Primer Pairs).

The raw sequencing outputs were filtered and trimmed for adapter sequences using skewer (55). The filtered and trimmed reads were checked for quality control using FastQC (https://www.bioinformatics.babraham.ac.uk/projects/fastqc/) and aligned to their respective genome sequence using Bowtie2 (56). The aligned sequences were visualized using Integrated Genomics Viewer (57).

### Analysis of metabolite extracts

Metabolites were extracted from cultures of *Streptomyces* sp. WAC07094 strains grown on Bennett’s agar or liquid medium. For agar-grown cultures (3 days of growth), agar (including cell biomass) was chopped into small pieces and soaked overnight in ethyl acetate (agar/biomass from two 10-cm plates in 50 mL). The organic solvent was evaporated under reduced pressure using a rotavap (Heidolph), and the resulting pellet was resuspended in 500 µL of methanol:acetone (1:1). The suspension was dried under reduced pressure and resuspended in 500 µL of a 1:1 mixture of methanol and water. For 4-day-old submerged cultures, 5 mL of cell-free broth was extracted using ethyl acetate at a 1:1 ratio. The organic phase was then removed and evaporated under reduced pressure using a speedvac (Thermo Scientific). The resulting pellets were dissolved in 50-µL dimethylsulfoxide (DMSO). After the removal of particulates by centrifugation, the extracts were subjected to LC-MS analyses under UV and ion detection.

Two approaches were used for saquayamycin detection and verification. The first involved analytical high performance liquid chromatography (HPLC) using an Agilent 1290 Infinity LC System with a Zorbax Eclipse XDB C18 column (100 mm × 2.1 mm × 3.5 µm, respectively), coupled to an LTQ Orbitrap XL MS system (Thermo Scientific). Metabolites were separated at a flow rate of 0.4 mL/min using a 2.5-min solvent gradient, from 95% solvent A (water with 0.1% formic acid) and 5% solvent B (100% acetonitrile) to 75% A and 25% B, a 10-min gradient to 5% A and 95% B, a 3-min isocratic under 5% A and 95% B. High resolution electrospray ionisation mass spectrometry (HR-ESI-MS) analysis was conducted under negative scan mode over a mass range of 100–2,000 Da. In the second, LC-MS was performed using an Agilent 1260 II Prime LC System with a Zorbax Eclipse XDB C18 column (100 mm × 2.1 mm × 3.5 µm, respectively), coupled to a 6475 triple quadrupole MS system. Metabolites were separated at a flow rate of 0.6 mL/min using a 2-min solvent gradient, from 70% solvent A (water with 0.02% acetic acid, 2-mM ammonium acetate, and 0.1-mM ammonium fluoride) and 30% solvent B (100% methanol) to 40% A and 60% B, a 10-min gradient to 20% A and 80% B, and 1-min final gradient to 2% A and 98% B. Electrospray (jet stream) was conducted under negative scan mode over a mass range of 350–850 Da, followed by product ion and multiple reaction monitoring (MRM) modes for *m*/*z* 819.3, *m*/*z* 559.1, and *m*/*z* 494.9 using settings of 100-V fragmentor and 30-V collision energy. The masses represented saquayamycin A/B and two unique product ions (qualifiers), respectively.

### Transcriptional reporter systems

A promoterless superfolder green fluorescence protein-encoding gene (with a terminator sequence upstream of the promoter cloning site to prevent read-through transcription) was amplified from pMC280 using primers pMS82MCS-F/pMS82MCS-R with a 60°C–64°C gradient annealing temperature. The resulting amplicons were cloned into the unique *Eco*RV site of the integrative plasmid pMS82 (58), yielding pMC281. The promoter region of the transcriptional activator *sqnR* (~0. 3kb) was amplified using primers PsqnR-up1/PsqnR-up2 (62°C–68°C annealing temperature) and was cloned in the *Hin*dIII site upstream of the promoterless green fluorescence protein-encoding gene. The resulting construct was verified by restriction analysis and sequencing using the universal M13-Reverse primer and was designated pMC282. This *sqnR* transcriptional reporter plasmid was then introduced into the WAC07094 Lsr2 knockdown strain where it integrated into the phiBT1 site in the chromosome.

We created an analogous transcriptional reporter construct for the *mtpA* gene. For this, we excised the *sqnR* promoter sequence from pMC282 and replaced it with the *mtpA* promoter. The *mtpA* promoter (~0.2 kb) was amplified using primers PmtpA-HindIII/PmtpA-MfeI with a gradient annealing temperature from 66°C to 70°C. The resulting reporter construct (pMC283) was confirmed by restriction analysis and sequenced using primer PmtpA-MfeI, before being introduced into WAC07094 strains. A positive control reporter strain for transcriptional activity was created by cloning the ermE* promoter upstream from the promoterless *gfp* gene and introducing this construct into different WAC07094 strains.

To visualize fluorescence for the different transcriptional reporter strains, the strains were inoculated onto solid growth medium and grown for 3 days before the colonies were imaged using a laser scanner (Typhoon FLA 9500) with an enhanced green fluorescent protein (EGFP) filter setting (473 nm). The resulting images were recorded without any further image processing.

### Creating deletion and complementation strains for *mtpAphoURP*

To simultaneously delete the *mtpA* and *phoURP* genes, DNA fragments flanking the four genes were amplified using primers pho-upKpnI/mtpA-dHindIII and pho-downSpeI/pho-downNotI with a 66°C–72°C gradient annealing temperature. A 3.1-kb fragment (upstream of the four genes) was cloned into pIJ2925, whereas a 3.3-kb fragment (downstream of the four genes) was cloned together with an apramycin resistance cassette (excised from pIJ773 using *Hin*dIII/*Spe*I) in pIJ777 (59). The downstream-flanking sequence and the apramycin resistance cassette were collectively excised using *Hin*dIII-*Psi*I and were introduced adjacent to the upstream flanking sequence at the *Hin*dIII-*Ssp*I sites. After construct integrity was confirmed by sequencing and digestion, the resulting plasmid (pMC284) was conjugated into WAC07094, and apramycin-resistant exconjugants were selected for. Exconjugants were screened for double crossover events by PCR using primers phoP-INchk/phoR-INchk (where no product was expected in the event of a successful deletion; wild-type chromosomal DNA was used as a positive control for these PCR checks) and oriT-2/mtpA-OUTchk with 65°C and 58°C annealing temperatures, respectively.

To dissect out the relative contribution of the four genes to the phenotype of the deletion mutant, four complementation constructs were created: (i) *mtpAphoURP*, using primers EXmtpA-PacI/pho-downKpnI; (ii) *mtpAphoU*, using primers EXmtpA-PacI/PphoR-downKpnI; (iii) *mtpA*, using primers EXmtpA-PacI/phoU-downKpnI; and (iv) *phoURP*, using primers PhoU-downPacI/pho-downKpnI. These amplicons were cloned into pIJ10257 (60) at the *Pac*I-*Kpn*I site (PermE* was excised out during cloning). The resulting constructs (pMC285–288, respectively) were introduced into the *mtpAphoURP* mutant by conjugation.

### Generating gene overexpression strains for select pleiotropic regulators

To individually overexpress *glnR* and *mtrA*, their genes were amplified from the WAC07094 genome using primers EXglnR-NdeI/EXglnR-XhoI or EXmtr-NdeI/EXmtr-PacI and a 58°C–65°C gradient annealing temperature. The resulting products were cloned into the *Nde*I/*Xho*I sites (*glnR*) or *Nde*I/*Pac*I site (*mtrA*) of the integrating plasmid pIJ10257, giving pMC289 and pMC290. Sequence integrity was confirmed by sequencing, after which the constructs were mobilized into both WAC07094 wild-type and Lsr2 knockdown strains through conjugation. Overexpressing the constitutive *afsQ1*_D52E_ was achieved using the thiostrepton-inducible pIJ6902-based construct (25).

### Assessing saquayamycin production during liquid culturing

Dilutions of WAC07094 or CCESR44 spores (10^7^–10^3^ or 10^7^–10^2^ viable spores/mL, respectively) were inoculated into 5 mL of Bennett’s liquid medium in 28-mL universal bottles. The cultures were then shaken (200 RPM) at 30°C for 4 days. Metabolites were extracted from the cultures using equal volumes of ethyl acetate. The solvent was evaporated *in vacuo*, and the metabolites were resuspended in 50-µL DMSO. For the bioassays, 10 µL of metabolite extract was applied to the filter disks, as described above.

### Genetic manipulation of strain 3212.3 for Lsr2 activity modulation and saquayamycin biosynthetic gene disruption

To modulate Lsr2 activity in CCESR44, the Lsr2 knockdown construct was introduced via intergeneric conjugation. Approximately 10^8^ viable spores were mixed with 1.5 mL of overnight cultures of *E. coli* ET12567/pUZ8002, before being spread on ISP4 agar supplemented with 20-mM MgCl_2_. After overnight growth, plates were overlaid with apramycin to select for exconjugants.

To disrupt saquayamycin biosynthesis in strain 3212.3, we took advantage of the fact that the *sqnHI* sequence was 98% identical (over 2.3 kb) between WAC07094 and CCESR44. We introduced the pIJ10700 + *sqnHI* plasmid (pMC341) into CCESR44 through conjugation, selecting for hygromycin-resistant exconjugants.

## Data Availability

The contig sequences of the *Streptomyces* WAC07094 genome have been deposited in DDBJ/ENA/GenBank under accession number JAVMJZ000000000. The version described in this paper is version JAVMJZ010000000. The genome sequence of *Streptomyces* sp. 3212.3 (isolate CCESR44) is also available through Genbank, under the accession number QTTM01000001.1.

## SUPPLEMENTAL MATERIAL

Supplemental Figures msystems.01368-23-s0001.pdf Fig. S1 to S8.

Supplemental Tables msystems.01368-23-s0002.xlsx Tables S1 to S4.

ASM does not own the copyrights to Supplemental Material that may be linked to, or accessed through, an article. The authors have granted ASM a non-exclusive, world-wide license to publish the Supplemental Material files. Please contact the corresponding author directly for reuse.
