# Supplementary material for: Multifactorial genetic control and magnesium levels govern the production of a Streptomyces antibiotic with unusual cell density dependence
Source: mSystems. 2024 Mar 11;9(4):e01368-23. doi: 10.1128/msystems.01368-23 (PMC11019849; doi:10.1128/msystems.01368-23)
Supplement: Supplemental Figures — Fig. S1 to S8. [file msystems.01368-23-s0001.pdf]

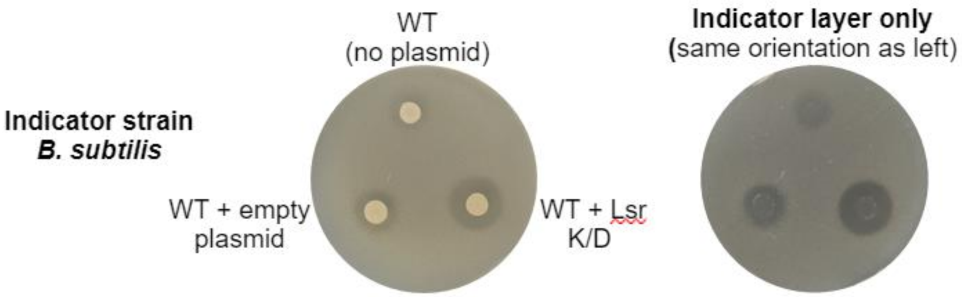

Figure S1. Antibiotic bioassay of *Streptomyces* WAC07094 strains. Antibiotic activity of wild type (WT) WAC07094 (top) was compared with that of an empty plasmid-carrying variant (left) and one carrying an Lsr2 knockdown (K/D)-expressing construct (right) using a ‘sandwich-based’ bioassay, where equal numbers of spores for each strain were spotted to Bennett’s medium, grown for 3 days, and then overlaid with nutrient agar infused with the indicator strain *Bacillus subtilis* (left image). The image to the right shows only the *B. subtilis*-infused nutrient agar (indicator layer) after being separated from the 'sandwich’ to better illustrate the zones of inhibition.

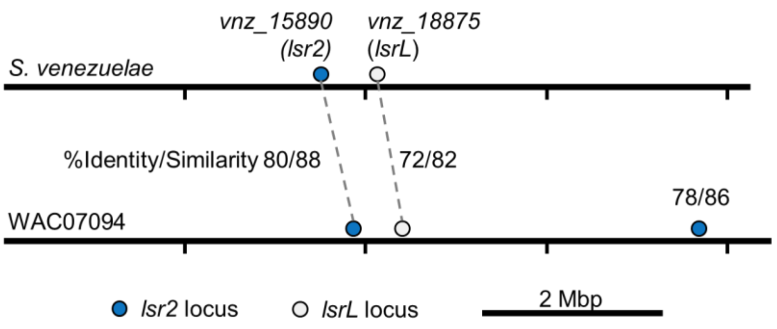

Figure S2. Relative locations and similarities of Lsr2 and its homologues in *S. venezuelae* and WAC07094. Schematic diagram illustrating the relative position of *lsr2* and *lsrL* homologues in the chromosomes of *S. venezuelae* and WAC07094. For WAC07094, the percent identity/similarity are indicated, relative to their *S. venezuelae* equivalents. For LsrS (the third Lsr2-like protein encoded by WAC07094), the identity/similarity values are relative to *S. venezuelae* Lsr2.

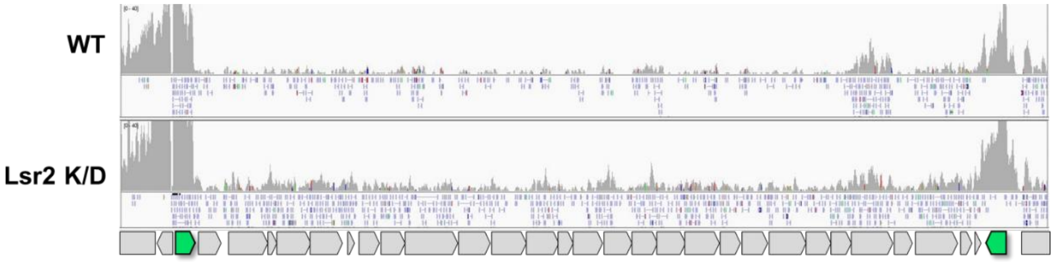

Figure S3. Relative transcript levels for the saquayamycin biosynthetic genes in wild type (WT) WAC07094 and the Lsr2 K/D strain. RNA was extracted from strains grown on Bennett's agar for 3 days. Transcript levels are represented by RNA sequencing read coverage (grey graphs). Representative sequencing reads are depicted in blue boxes under each graph, for genes oriented in the forward direction. Two regulatory genes (shown as green arrows) are located at either end of the gene cluster.

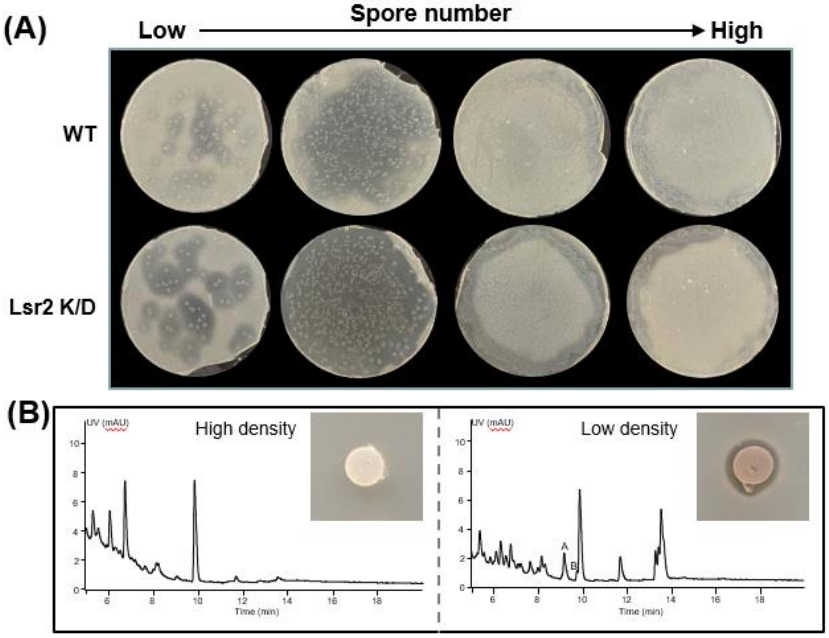

Figure S4. Impact of spore density on antibiotic activity of WAC07094. (A) Antibiotic bioassay of a spore dilution series for wild type (WT) and Lsr2 knockdown (K/D) strains against *B. subtilis.* **(B)** Chromatograms (UV) of crude extracts prepared from Bennett's agar from high-density (left) and low-density (right) Lsr2 knockdown strains grown for 3 days. Peaks corresponding to saquayamycin A and B are indicated with the A and B labels (respectively) within the chromatogram. Inset: anti-*Bacillus* assay using plugs taken from the centre of a confluent lawn (high density) or plugs taken from the centre of a spotted ~7 mm colony (6 spots/plate to represent a low density condition).

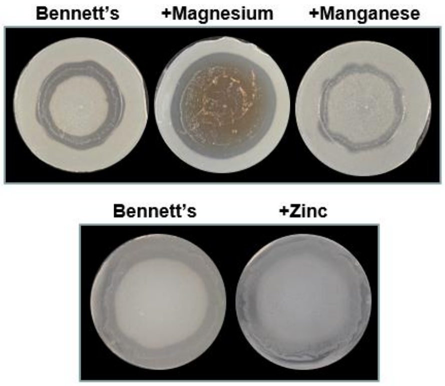

Figure S5. Effect of diverse metal ions on saquayamycin production. Antibiotic bioassay of the Lsr2 knockdown strain grown for 3 days on Bennett's agar alone, or supplemented with magnesium chloride, manganese chloride, or zinc chloride. Sensitive indicator strain is *B. subtilis*.

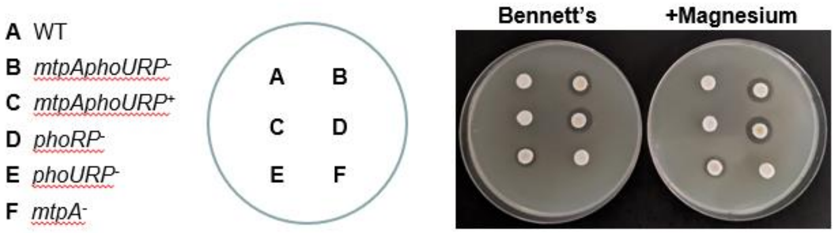

Figure S6. Loss of PhoRP can overcome the density-dependent production of saquayamycin during liquid culture growth. Antibiotic bioassays were conducted using extracts from high-density liquid cultures of WAC07094 (inoculated with 10^7 spores/mL) grown for 4 days in Bennett's liquid medium. Ten microlitres of extract from each of the six wild type and mutant cultures were applied to filter discs overlaid on medium mixed with *B. subtilis*.

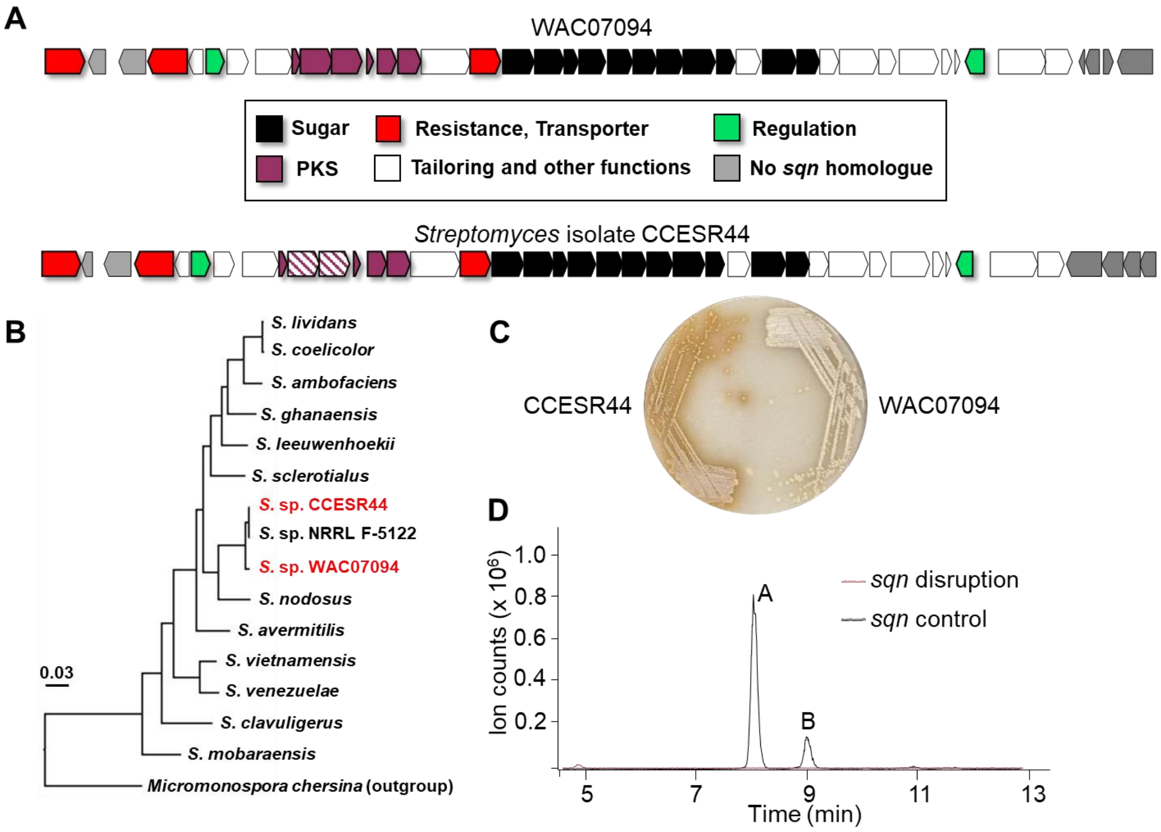

Figure S7. Comparison of saquayamycin producer strains WAC07094 and *Streptomyces* isolate CCESR44 (A) Saquayamycin biosynthetic clusters in WAC07094 and the alternative producer *Streptomyces* isolate CCESR44**.** Minor differences of gene organization are coloured in grey. Those biosynthetic genes targeted for disruption (by homologous recombination) are indicated in purple hatching. **(B)** Phylogenetic tree of strains WAC07094 and CCESR44 relative to other streptomycetes, using *Micromonospora chersina* as an outgroup. Bootstrap values of each branch: greater than or equal to 50% (based on 1000 resampled trials). The scale bar (labelled 0.03) represents substitutions per nucleotide position. The phylogenetic analysis was conducted using https://automlst.ziemertlab.com/index (26) based on 50 select single copy conserved genes. **(C)** Phenotypic comparison of WAC07094 and CCESR44 following growth on mannitol-soy flour agar. **(D)** Extracted ion chromatograms of $[M-H]^-$ (at *m/z* 819.3) from the extracts of the 3212.3 *sqnHI* mutant and WAC07094 (as a positive control) grown from low-density spore inoculum in Bennett's liquid medium for 4 days.

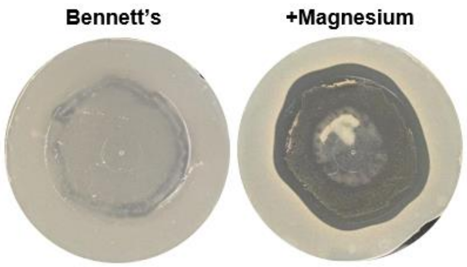

Figure S8. Magnesium supplementation can overcome the density-dependent saquayamycin production of strain 3212.3. Antibiotic bioassays using *B. subtilis* as the indicator strain inoculated into agar that was overlaid atop *Streptomyces* sp. CCESR44 grown on Bennett's agar for 3 days with and without magnesium supplementation.
